# RETRACTED: Khan et al. Bi and Sn Doping Improved the Structural, Optical and Photovoltaic Properties of MAPbI3-Based Perovskite Solar Cells. *Materials* 2022, *15*, 5216

**DOI:** 10.3390/ma18071430

**Published:** 2025-03-24

**Authors:** Muhammad I. Khan, Sumra Yasmin, Norah Alwadai, Muhammad Irfan, Ikram-ul-Haq, Hind Albalawi, Aljawhara H. Almuqrin, Maha M. Almoneef, Munawar Iqbal

**Affiliations:** 1 Department of Physics, The University of Lahore, Lahore 53700, Pakistan; sumrayasmin666@gmail.com (S.Y.); muhammad.irfan6252@gmail.com (M.I.); ikrmhaq@gmail.com (I.-u.-H.); 2 Department of Physics, College of Sciences, Princess Nourah bint Abdulrahman University, P.O. Box 84428, Riyadh 11671, Saudi Arabia; hmalbalawi@pnu.edu.sa (H.A.); ahalmoqren@pnu.edu.sa (A.H.A.); mmalmoneef@pnu.edu.sa (M.M.A.); 3 Department of Chemistry, The University of Lahore, Lahore 53700, Pakistan

*Materials* retracts the article, “Bi and Sn Doping Improved the Structural, Optical and Photovoltaic Properties of MAPbI3-Based Perovskite Solar Cells” [1], cited above.

Following publication, concerns were brought to the attention of the publisher regarding to the validity of the XRD data presented in this article [1].

Adhering to our complaints procedure, an investigation was conducted by the Editorial Office and Editorial Board. While the authors fully cooperated with the Editorial Office during the investigation, they were unable to satisfactorily explain the presence of a number of significant anomalies within the raw XRD data. As a result, the Editorial Board and Editor-in-Chief were unable to confirm the reliability of the findings and subsequently decided to retract this paper as per MDPI’s retraction policy (https://www.mdpi.com/ethics#_bookmark30) and in line with the Committee on Publication Ethics retraction guidelines (https://publicationethics.org/retraction-guidelines).

This retraction was approved by the Editor-in-Chief of the journal *Materials*.

The authors did not agree to this retraction.
